# Long-Lasting Chews Elicit Positive Emotional States in Dogs during Short Periods of Social Isolation

**DOI:** 10.3390/ani13040552

**Published:** 2023-02-04

**Authors:** Hannah E. Flint, Megan Atkinson, James Lush, Alysia B. G. Hunt, Tammie King

**Affiliations:** Waltham Petcare Science Institute, Freeby Lane, Waltham on the Wolds, Leicestershire LE14 4RT, UK

**Keywords:** dog, food-based enrichment, emotional wellbeing, behavior

## Abstract

**Simple Summary:**

Dogs are a social species and may experience negative emotions when left alone even for short periods of time. This study explored the effectiveness of different food-based enrichments on engaging dogs, and alleviating potential negative emotional states caused by social isolation. The results indicated that dogs spent the most time interacting with a long-lasting chew. They also responded to this enrichment with the most positive and calm emotions when compared to a treat-dispensing toy and a smart treat-dispensing device. Long-lasting chews should be further explored as an enrichment for improving dog emotional wellbeing during periods of separation from their caregivers.

**Abstract:**

Dogs may experience negative emotional states when isolated from human caregivers and conspecifics. This study aimed to evaluate how dogs interact with different enrichments during a short period of social isolation, as a first step towards identifying methods for improving their emotional wellbeing. Using a cross-over design, dogs (*n* = 20) at the Waltham Petcare Science Institute were exposed to four different food-based enrichments while left alone in a familiar room for 20 min: long-lasting chew (Chew), kibble in a treat-dispensing toy (Toy), and kibble dispensed through a smart treat-dispensing device with (Device + Voice) and without (Device) a person talking to the dog. Time spent engaging with each enrichment item and emotional valence and arousal (7-point scale collected every 5-min) were scored from videos. The results of linear mixed models indicated Chew was the most successful enrichment, with dogs having lower arousal scores (*p* < 0.05 vs. Device and Toy) during the first five minutes of isolation, higher positive valence scores (*p* < 0.05 vs. all) during the second five minutes of isolation, and spending the most total time engaged (*p* < 0.01 vs. all). Based on these findings, long-lasting chews should be further explored to assess their impact on dog emotional wellbeing.

## 1. Introduction

One of the major welfare concerns affecting pet dogs in modern society is negative emotional states caused by extended periods of separation from attachment figures such as owners or other pets. Dogs are a social species that can develop meaningful connections with their caregivers, similar to those between children and their parents [1]. When isolated for extended periods, such as when their owners are at work, dogs are likely at risk of negative emotional states, including boredom, frustration, fear and anxiety [2]. Separation-related problems, which can be defined as any “behavior that is a problem for an owner when their dog is left alone, regardless of cause” [2], are estimated to be shown by 22–55% of dogs [2]. It is possible a much larger proportion of dogs suffer from issues that are related to separation but are not visible to the owner. This may occur due to a lack of behavioral expression from dogs [3], owners not accurately recognizing signs of separation-related issues, or simply because the owner is not present when the behaviors are exhibited. This behavioral concern is particularly relevant due to recent events following the lockdowns of the COVID-19 pandemic. Many dogs spent more time in close contact with their caregivers, and owners have reported being concerned that their pets will develop separation anxiety as people start returning to their normal routines [4]. Overall levels of separation anxiety in the United Kingdom do not appear to have changed between the start of the lockdown period and October 2020 [5]. However, a subset of dogs who had not previously shown signs of separation anxiety before the pandemic started exhibiting separation-related behavioral problems by October 2020, with those who had the greatest reduction in time left alone being the most at risk [5]. Additionally, many workplaces had not fully returned to normal working by October 2020, so it is possible additional increases in separation-related problems will be seen in coming months and years.

For severe cases of separation anxiety, strict desensitization and counter-conditioning protocols, aimed at altering the dog’s emotional reaction to the owner’s departure, have been demonstrated to be effective in improving behavior [6]. In addition, numerous studies have examined the efficacy of different anti-anxiety medications such as clomipramine [7,8] and fluoxetine [9,10], or more “natural” remedies such as dog-appeasing pheromone [7], which can be used in combination with the behavior modification protocols to assist in increasing the latter’s efficacy. Numerous studies have been conducted assessing the efficacy of these treatment plans with varying degrees of success [11]. However, these protocols require significant investment of time, dedication and skill level from the owner, as well as financial costs of consulting a behavior professional [12]. Additionally, these interventions may not be as effective for separation-related behaviors motivated by emotional states other than anxiety, such as frustration or boredom when left on their own [2].

An alternative method for alleviating negative emotional states and improving animal welfare is through environmental enrichment. Enrichments can take many forms, including changes to the physical structure of the environment, providing different sensory stimulation, or through different feeding methods [13]. Generally, the aim of enrichment is to add complexity to the animal’s environment, which can result in improved welfare by providing the animal with choices of different behaviors they can perform, leading to increased behavioral diversity. Successful enrichment can result in a reduction in abnormal behaviors while increasing positive interactions with the environment, promoting a positive emotional state. Enrichments can also make more permanent changes to the animals’ wellbeing through improved ability to cope with challenges and improved cognitive performance [13,14]. While the use of various types of enrichments to improve animal welfare has been extensively studied in captive animals in zoo [15,16] and farm settings [17,18], less research has studied enrichments for companion animals. Research on dogs to date has primarily been limited to populations of kenneled dogs in laboratory or shelter settings [19]. For example, studies in laboratory-housed dogs have demonstrated that provision of chew toys was successful in decreasing inappropriate chewing and time spent inactive [20], while the provision of a treat-stuffed toy contributed to more variable behavioral patterns, including decreased inactivity and barking and increased appetitive behaviors [21]. In shelter dogs, enrichment provided in the form of training sessions and food-filled toys increased sitting, lying, and quiet behavior, as well as decreased jumping [22], while the provision of classical music resulted in more time sleeping and less time vocalizing [23]. A study examining assistance dogs identified that enrichments were successful in increasing relaxation behaviors and reducing alert and stress behaviors in the hour after the enrichment was provided [24]. This effect was greatest with play-based enrichments and lowest in food-based enrichments. However, the play-based enrichments used in that study required social interaction with another dog or handler and, therefore, would not be possible during periods of social isolation. Another study which investigated pet dogs found that the provision of food-based enrichments successfully reduced problematic behaviors when dogs were separated from their owners for a short period of time [25]. While dogs were not socially isolated during that study as the experimenter remained in the room, this does indicate food-based enrichments may be a promising option for alleviating negative emotional states in dogs during periods of social isolation.

Pet dogs often receive enrichment through interactions with their owners; however, this form of enrichment is no longer present when dogs are left alone. While it is commonly recommended by professionals that dogs be provided with different types of enrichment when being left alone for extended periods, what type of enrichments to use and the efficacy of these interventions have not been scientifically evaluated.

The overall aim of this study was to identify which forms of enrichment, in this case treats and treat-filled devices, dogs chose to interact with when left alone. The secondary objective was to determine what effect these different enrichments had on the dog’s emotional state.

## 2. Materials and Methods

### 2.1. Subjects

Twenty adult dogs, twelve females and eight males (two entire females and one entire male), representing a medium breed (10 Petit Basset Griffon Vendéens) and a large breed (10 Labrador Retrievers), with a mean age (±sd) of 4.8 ± 2.5 years ranging from 1.6 to 9.7 years, participated in the study. All dogs were housed in pairs or small groups within kennels at the Waltham Petcare Science Institute (Leicestershire, UK), with housing kept consistent throughout the duration of the study. All dogs were provided with comprehensive training and socialization programs, adjusted to the needs of individual dogs as per the institute’s standard husbandry requirements. All dogs were routinely fed two meals per day with a morning feed at 9:30 a.m. and an afternoon feed between 2:30 and 3:00 p.m.

In the interest of dog and human welfare, dogs were excluded based on previous observations of resource guarding, or excessive destructive behavior with risk of self-injury or ingestion of foreign bodies, as well as any dietary restrictions that would not allow the consumption of the treats or kibble used during testing. Prior to the commencement of testing, all dogs were habituated to the test room while in the presence of a familiar handler. In addition, each dog was exposed to each of the enrichments while in the presence of a familiar handler to ensure acceptance and to minimize the effect of any differences in previous experience. The duration and frequency of these habituations and exposures were not defined. Instead, a minimum of one exposure was required, with additional sessions occurring as needed until the dog displayed confident body language and interacted with the enrichments. An average of 2.25 sessions were required to reach habituation criteria, with all dogs reaching criteria within 3 sessions (15% 1 session; 45% 2 sessions; and 40% 3 sessions).

Once fully comfortable in the testing environment, each dog was exposed to a social isolation event, where they were left alone in the room for 20 min. During this first exposure, no enrichments were present, and no data were collected. This event served to reduce potential novelty effects on the dog’s responses.

### 2.2. Testing Area

**Figure 1:**
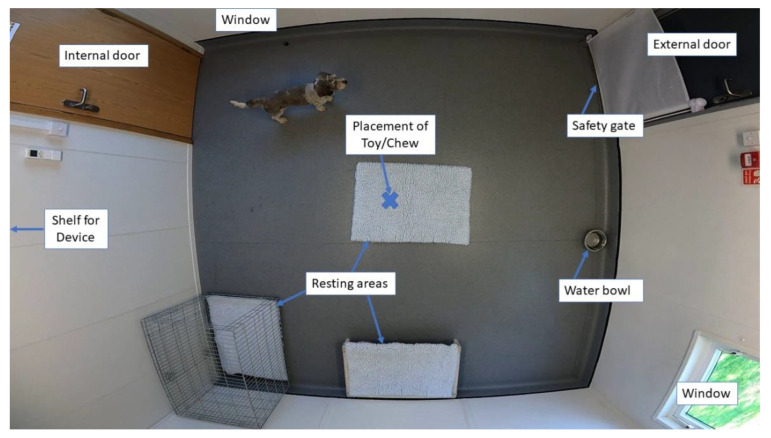
Test room setup used for social isolation events.

All testing was conducted within a test room (3.71 m × 3.58 m) located within a portable building at the Waltham Petcare Science Institute (Figure 1). The room had two windows and an internal and external door (only the external door was used to bring dogs into and out of the room). In the interest of dog safety, a retractable mesh safety gate was mounted inside the external door and was used to prevent the dog rushing out when the handler opened the door upon returning. The room was set up in a standardized manner, with multiple resting areas, including vet bedding placed on the floor, in a crate, and on an elevated dog bed. Vet bedding was changed between each dog and the room cleaned with a virucidal cleaner (Selgeine, Selden Research Ltd., Buxton, UK). In addition, dogs were provided with free access to fresh water in the form of a metal water bowl that was filled at the beginning of each test session. A radio played in an adjoining room throughout the test sessions to mask potential background noises that may have distracted the dogs.

### 2.3. Enrichments

Four food-based enrichments were selected to be tested to represent different types of enrichment recommended for use when leaving dogs home alone. These were a long-lasting chew (Chew), a food-dispensing toy (Toy), and a smart food-dispensing device with (Device + Voice) and without (Device) a person talking to the dog via the device’s app. For the Chew enrichment session, a chew (PEDIGREE^®^ GOOD CHEW^TM^ Treat, Mars Petcare UK, Slough, UK) was placed in the center of the test room at the start of the session, with the dogs over 25 kg receiving a large-sized chew and dogs 25 kg and under receiving a medium-sized chew (as per recommended feeding guidelines). Adult dog main meal kibble (JAMES WELLBELOVED^TM^ Adult Lamb and Rice, Mars Petcare UK, Slough, UK) was used for the Toy and Device; it was selected to be rewarding to the dogs based on novelty and palatability and had a consistent size and shape (~11 mm diameter) that enabled it to be obtained from the Toy and minimized jamming in the Device. The amount offered was 77 g for medium dogs (≤25 kg) and 126 g for large dogs (>25 kg). This amount was selected to match the kcal of the respective chews (medium: 270 kcal, large: 445 kcal). For the Toy enrichment session, a food-dispensing toy (KONG Wobbler^TM^, KONG Company EU Ltd., Wiltshire, UK) was filled with the appropriate quantity of kibble and was left in the center of the room, except for two dogs who refused the novel kibble and who were instead given their normal background dry main meal diet. The toy was hollow and made of a hard, durable plastic with a weighted base, and had a small round hole at the side through which kibble could fall out. The dogs were required to move the toy with their paw or nose to cause it to rock and kibble to fall from the hole. For both the Device sessions, a smart food-dispensing device (FURBO^TM^, Taipei, Taiwan) was filled with kibble and placed on a high shelf (~1.8 m) within the test room out of reach of the dog. The experimenter used an app on a mobile phone or tablet to trigger the device to dispense kibble once every minute during the social isolation event. Furthermore, for the Device + Voice sessions, a script was read by the experimenter (female) using a friendly, high-pitched voice (as they would typically talk to a dog they were trying to engage) that consisted of a series of positive phrases (Appendix A—Device Script). One of these phrases was read immediately prior to dispensing each kibble.

### 2.4. Study Design

Dogs were exposed to each of the four different food-based enrichments separately during a series of 20 min social isolation events in a cross-over design with order randomized using a balanced Latin Square. All testing occurred from April to July 2021, with sessions scheduled in the afternoon prior to their afternoon meal (between 12:30 and 2:30 p.m.). Each dog participated in a maximum of two sessions a week with at least one day in between test sessions. On the days dogs received enrichment, their daily food was reduced by 25% to avoid weight gain, and any food not consumed during the sessions was re-offered at their afternoon meal.

Dogs were closely monitored throughout each test session using CCTV cameras (Dahua 4K IR Turret Network Camera with built-in microphone and varifocal motorized lens: 2.7–12 mm) and were monitored for signs of distress and/or safety concerns based on pre-defined removal criteria. These included excessive barking, hyperventilation, extreme hypersalivation, cowering, repeated vigorous escape attempts, and behaviors resulting in self-harm and/or the ingestion of foreign bodies. While no dogs had to be removed from test sessions during the study, one dog was removed from the study following three test sessions due to a deterioration of behavior across repeated testing (1.8yo entire male Labrador Retriever). This included a general increase in undesired behaviors observed outside of testing, as well as an increase in negative responses observed during the test sessions, including poor recovery upon return of the handler. The data from this dog were removed from analysis.

### 2.5. Data Collection

During each social isolation event, dogs were video-recorded using the CCTV cameras mounted in each corner of the room. Two camera angles that covered the entirety of the room were used for behavioral coding. The duration and/or frequency of different dog behaviors were coded by two trained coders using The Observer XT (version 15) following a detailed ethogram (Table 1). Videos were randomly assigned to coders, with 10 videos coded in common to assess inter-rater reliability. Additionally, 5 videos were re-coded by each coder for a total of 3 repetitions to assess intra-rater reliability. The first repetition from the assigned coder for each video was used for the subsequent analyses so there was only one score per video.

In addition, four trained coders scored all videos on a series of adjectives using a Qualitative Behavioral Assessment (QBA) previously developed to evaluate welfare in shelter dogs [26] with modifications to make the tool more relevant to the study objective and context of testing. This included the addition of the terms ‘calm’, ‘engaged’, ‘frustrated’, ‘lethargic’, ‘restless’, ‘sad’, ‘tense’, and ‘uncomfortable’, and the removal of the terms ‘aggressive’, ‘attention-seeking’, ‘sociable’, and ‘wary’ for a total of 24 terms (Table 2). Coders provided a score for each term for every 5-minute interval (0 to 5, 5 to 10, 10 to 15, and 15 to 20) during the social isolation event. A visual analog scale from 0 to 125 mm was used, with the lowest score (0) meaning the quality indicated by the term was absent in the dog, and the highest score (125) meaning the quality indicated by the term was strongly dominant in the behavior of the dog. Finally, each coder rescored all timepoints for 5 videos (20 observations) to determine intra-rater reliability.

At the same time as they were scoring the QBA, the four coders also provided scores for emotional valence (the extent to which an emotion is positive or negative) and arousal (emotional intensity) on scales from 1 (very negative valence/not arousing) to 7 (very positive valence/highly arousing) [27].

### 2.6. Statistical Analysis

Intraclass correlation coefficients (ICCs) using two-way mixed effects models were calculated to assess reliability for all measures [28]. Consistency agreement was used for inter-rater reliability, and absolute agreement was used for intra-rater reliability [28]. These values were interpreted as poor (ICC < 0.50), moderate (ICC: 0.50–0.75), good (ICC: 0.75–0.90) or excellent (ICC > 0.90) [28].

Mean scores across the coders were generated for the QBA terms and the valence and arousal scales. The QBA terms were then summarized using a principal components analysis (PCA). Prior to analysis, the data were assessed for appropriateness of inclusion in a PCA using a Kaiser–Meyer–Olkin (KMO) measure of sampling adequacy and Bartlett’s test of sphericity [29]. The data met the requirements for a PCA with KMO values >0.50 (overall KMO = 0.88) and a significant Bartlett’s test (*p* < 0.001) [29]. For interpretation of the components, terms with loadings ≥|0.50| were considered to provide a significant contribution to the component. Each term’s weighting from key components identified during this analysis was used to generate component scores. These component scores were then evaluated for inter- and intra-rater reliability using ICCs as described above to determine the reliability at the level of the component [30].

QBA component scores, emotional valence and arousal scales, and durations of state behaviors were analyzed using linear mixed-effect models with Intervention (Chew vs. Toy vs. Device vs. Device + Voice) as the fixed effect and Dog and Order as random effects. Time (5 min interval), Intervention, and the interaction between Time and Intervention were included as fixed effects for the QBA and emotional scale analyses. Breed, age, and the number of habituation sessions required to reach confidence were tested as possible covariates but were nonsignificant in all analyses and, therefore, were removed from the models. A random effect of Observer was tested for the coded behavior models but was removed from all except other stationary behavior due to singular fit. Additionally, the random effect of Order was removed from engaged without contact and other locomotion. All models were checked for fit by visual inspection of the residuals and were log-transformed where appropriate. Estimated means (back-transformed if appropriate) for each intervention were plotted with 95% confidence intervals. All pairwise comparisons between interventions (and time where appropriate) were analyzed and *p*-values for significant effects (*p* < 0.05) reported. Escape and destructive behaviors were not analyzed as durations as they occurred in fewer than 50% of observations. Instead, they were analyzed as yes/no for occurrence. For these behaviors, a binomial-generalized linear mixed-effects model was used, with Intervention as the fixed effect and Dog and Order as random effects. The random effect of Observer was tested in the models but removed for both behaviors due to singular fit. The estimated probability of dogs performing the behavior for each intervention was plotted with 95% confidence intervals. All pairwise comparisons between interventions were analyzed and *p*-values for significant effects (*p* < 0.05) reported. The behaviors of elimination, shake-off and autogrooming could not be analyzed due to infrequent occurrence (<20% of observations).

All analyses were performed using R version 4.0.4 [31].

## 3. Results

### 3.1. Coded Behaviors

Inter-rater reliability analysis demonstrated there was excellent agreement for duration of engaging with contact (ICC: 1.00), engaging without contact (ICC: 0.95), other active behavior (0.99), other stationary behavior (1.00), and escape behavior (0.99), and moderate agreement for the duration of destructive behavior (ICC: 0.56). Autogrooming, elimination, and shake-off did not occur in the reliability videos, and therefore could not be analyzed. For intra-rater reliability there was excellent agreement for all the behaviors that were present for both raters (ICC: 0.90–1.00). The coding for these behaviors was, therefore, deemed sufficiently reliable for analysis.

**Figure 2:**
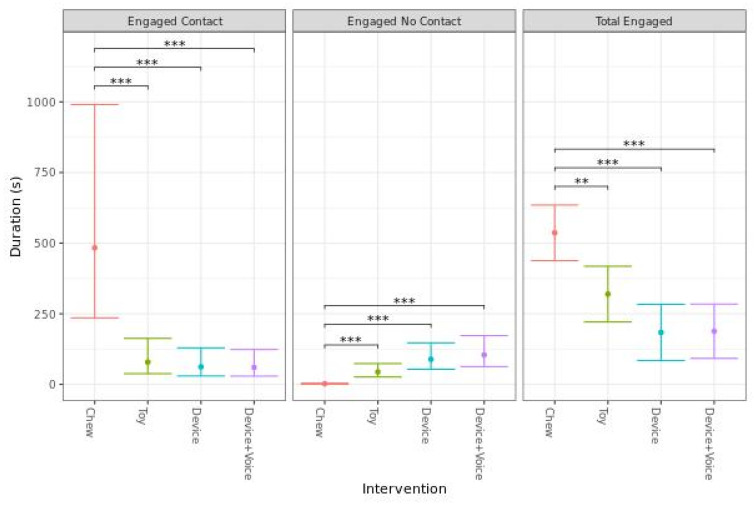
Predicted mean (95% CI) duration of time (s) dogs spent engaged with physical contact, engaged without physical contact, or total engagement (sum of engaged with and without physical contact) when left alone with one of four enrichments for 20 min. Asterisks indicate significant differences between treatment groups. Three asterisks indicate significance at *p*  <  0.001, two asterisks indicate significance at *p*  <  0.01.

The duration of engagement with each enrichment item was analyzed in three ways: engaged with contact, engaged without contact, and total engagement (sum of with and without contact). Due to a lack of homoscedasticity in the residuals, a log transformation was applied to the model for engaged with contact and engaged without contact. The model for total engagement met model assumptions and proceeded without transformation. The results of these analyses indicated dogs spent significantly more time engaged with contact for the Chew compared to any other intervention (*p* < 0.001). There were no significant differences in time engaged with contact between the Toy, Device, or Device + Voice interventions. Conversely, dogs spent significantly less time engaged without contact for the Chew compared to any other intervention (*p* < 0.001), with no significant differences between the other interventions. Finally, dogs spent significantly more total time engaged for the Chew compared to the Toy (*p* = 0.002), Device (*p* < 0.001), or Device + Voice (*p* < 0.001) with no significant difference between the other interventions. The predicted mean [95% CI] total time engaged was 8 min 57 s [7 min 18 s, 10 min 35 s] for the Chew, 5 min 20 s [3 min 41 s, 6 min 58 s] for the Toy, 3 min 4 s [1 min 25 s, 4 min 43 s] for the Device, and 3 min 8 s [1 min 32 s, 4 min 434] for the Device + Voice (Figure 2). There were only four occasions where dogs failed to meaningfully engage with the enrichments (defined as total time engaged <30 s), all of which occurred with the Toy.

**Figure 3:**
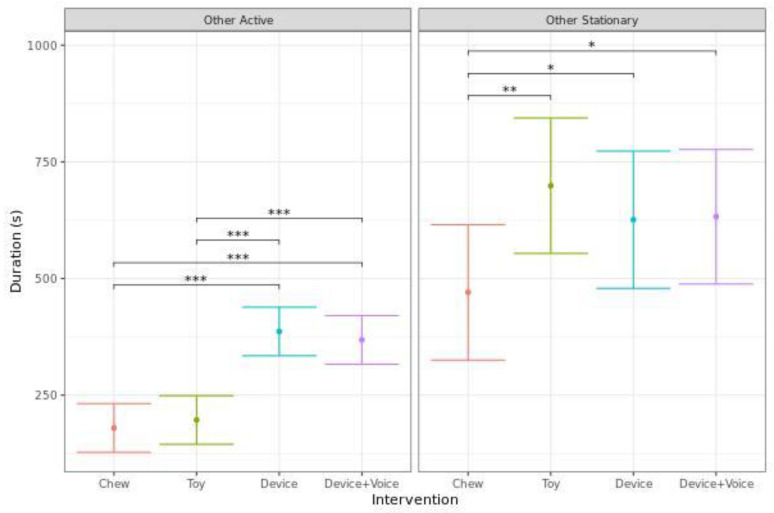
Predicted mean (95% CI) duration of time (s) spent performing other active behaviors, or other stationary behaviors when dogs were left alone with one of four enrichments for 20 min. Asterisks indicate significant differences between treatment groups. Three asterisks indicate significance at *p*  <  0.001, two asterisks indicate significance at *p*  <  0.01, and one asterisk indicates significance at *p*  <  0.05.

Dogs spent significantly more time performing other active behaviors for both Device sessions in comparison to either the Chew or Toy (*p* < 0.001). However, there were no significant differences in other active behaviors between the Devices, or between the Chew and Toy. Dogs spent significantly less time performing other stationary behaviors for the Chew compared to the Toy (*p* < 0.001), Device (*p* = 0.036), or Device + Voice (*p* = 0.027), with no significant differences between the other interventions (Figure 3).

**Figure 4:**
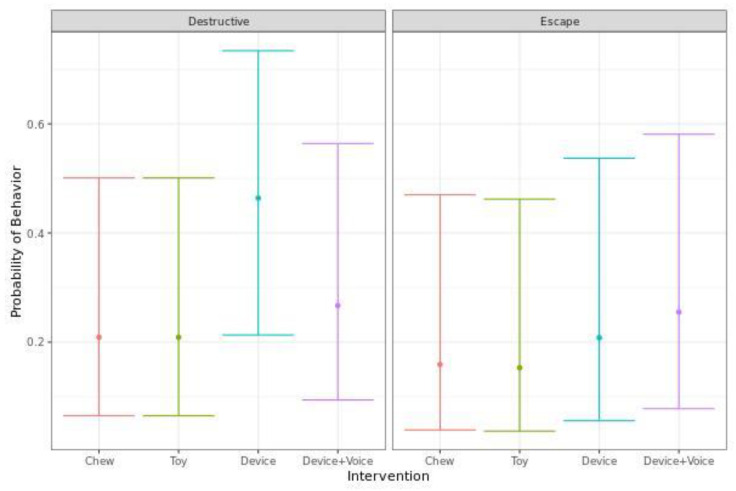
Predicted probability (95% CI) of dogs performing destructive or escape behaviors when they were left alone with one of four enrichments for 20 min. No significant differences between treatments groups were identified( *p*  <  0.05).

There were no significant differences in the probability of escape or destructive behavior based on intervention (Figure 4).

**Figure 5:**
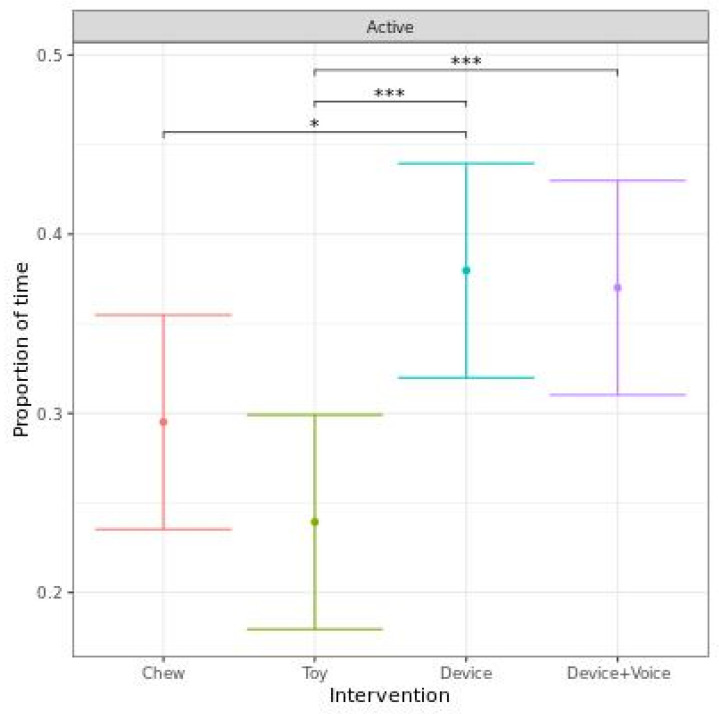
Predicted proportion (95% CI) of time spent active when “not engaged” when dogs were left alone with one of four enrichments for 20 min. Asterisks indicate significant differences between treatments groups. Three asterisks indicate significance at *p*  <  0.001, and one asterisk indicates significance at *p*  <  0.05.

When analyzed as the proportion of time spent active when “not engaged”, dogs spent a significantly higher proportion of time active for the Device compared to the Chew (*p* = 0.029) and Toy (*p* < 0.001) and for the Device + Voice compared to the Toy (*p* < 0.001). There was a tendency for the Device + Voice to have a higher proportion of time active compared to the Chew (*p* = 0.064), but there was no significant difference between the Chew and Toy or between the two Devices (Figure 5).

### 3.2. Qualitative Behavior Assessment (QBA)

Inter-rater reliability analysis demonstrated that agreement was generally moderate (ICC: 0.50–0.75) for a majority of the QBA terms, with interested, engaged, frustrated, playful, alert, and explorative having poor agreement (ICC: <0.50) and only fearful having good agreement (ICC: 0.75–0.90). Intra-rater reliability was variable, with agreement ranging from none to perfect agreement depending on the term and coder (Table 3).

Based on these reliability results, Coder 4 was removed from the analysis of QBA as they had poor intra-rater reliability for several terms. In addition, the terms ‘alert’, ‘bored’, ‘depressed’, ‘excited’, ‘fearful’, ‘interested’, ‘lethargic’, ‘nervous’, ‘playful’, ‘reactive’, and ‘tense’ were not included in further analyses as they had poor intra-rater reliability for at least one of the remaining coders. Finally, an average score of all three remaining coders was used for analysis in order to minimize the impact of inter-rater variation. With these modifications, the remaining terms were considered sufficiently reliable for further analysis.

**Figure 6:**
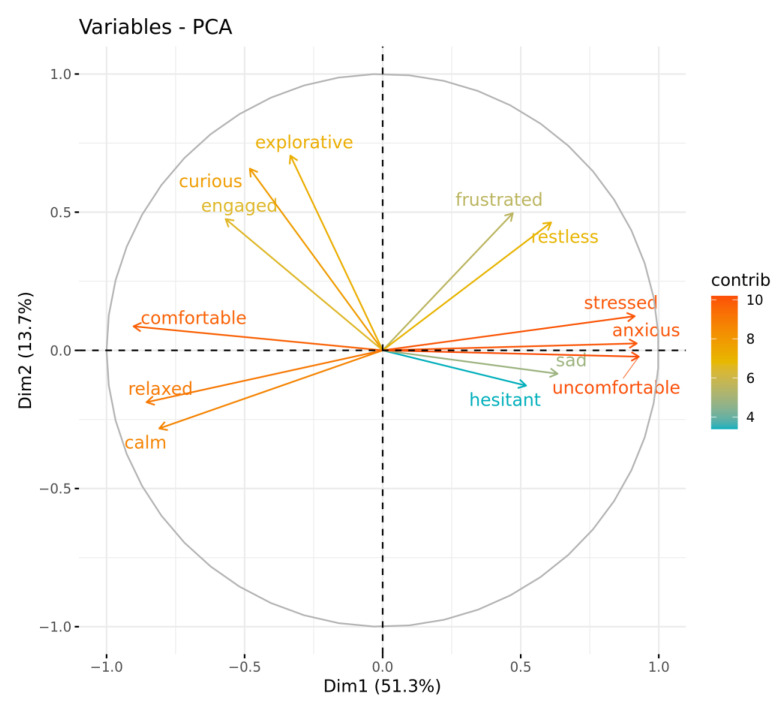
Plot of component loadings generated from a PCA of QBA terms showing the two main components of interest (Dim1: Stressed/Anxious, Dim2: Interactive) with arrow color indicative of the strength of contribution of the term.

Analysis of the QBA data using a PCA suggested two main components of interest based on the strength of loadings and the variance explained (Table 4; Figure 6). The first component explained 51.3% of the total variance and was labelled ‘Stressed/Anxious’. It was comprised of positive loadings for the terms ‘uncomfortable’, ‘anxious’, ‘stressed’, ‘sad’, ‘restless’, ‘and ‘hesitant’, and negative loadings for the terms ‘comfortable’, ‘relaxed’, ‘calm’, and ‘engaged’. The second component explained 13.7% of the total variance and was labelled ’Interactive’. It was comprised of positive loadings for the terms ‘frustrated’, ‘explorative’, and ‘curious’.

Results of reliability analysis of the component scores indicated that inter-rater reliability was moderate for both the ‘Stressed/Anxious’ (ICC: 0.69) and ‘Interactive’ (ICC: 0.59) components. Additionally, intra-rater reliability was good to excellent for all raters for both the ‘Stressed/Anxious’ (ICC: 0.86–0.99) and ‘Interactive’ components (ICC: 0.85–0.99).

**Figure 7:**
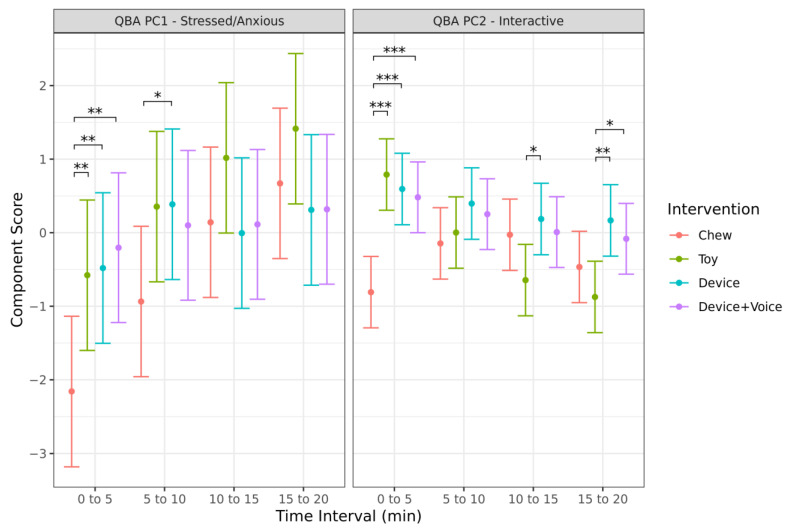
Predicted mean (95% CI) QBA ‘Stressed/Anxious’ and ‘Interactive’ component scores for each 5 min interval when dogs were left alone with one of four enrichments for 20 min. Asterisks indicate significant differences between treatment groups within each time interval. Three asterisks indicate significance at *p*  <  0.001, two asterisks indicate significance at *p*  <  0.01, and one asterisk indicates significance at *p*  <  0.05.

There was a significant interaction between time and intervention for the ‘Stressed/Anxious’ component score (*p* = 0.025; Figure 7). Pairwise comparisons identified that the Chew had significantly lower ‘Stressed/Anxious’ component scores (indicating more positive emotional states) compared to the Toy (*p* = 0.009), Device (*p* = 0.005), and Device + Voice (*p* = 0.001) interventions during the first five minutes, and also had significantly lower scores compared to the Toy (*p* = 0.050) and Device (*p* = 0.043) interventions during the second 5 min. There were no other significant differences between the interventions at any timepoint. Both the Device interventions had no significant changes over time. The Chew and Toy did not have significant changes in ‘Stressed/Anxious’ component scores between any two consecutive time intervals; however, there was a significant increase in the ‘Stressed/Anxious’ component score from the first five minute interval to the last five minute interval for both interventions (*p* < 0.001), indicating an increase in negative emotions over time.

There was a significant interaction between time and intervention for the ‘Interactive’ component score (*p* < 0.001; Figure 7). Pairwise comparisons identified that the Chew had significantly lower ‘Interactive’ component scores compared to the Toy (*p* < 0.001), Device (*p* < 0.001), and Device + Voice (*p* < 0.001) interventions during the first five minutes. Conversely, the Toy had significantly lower ‘Interactive’ component scores than the Device (*p* = 0.016) intervention during the third five minutes and lower ‘Interactive’ component scores than the Device (*p* = 0.001) and Device + Voice (*p* = 0.026) interventions during the last five-minute interval. Only the Toy intervention had significant changes over time, with the score significantly decreasing from the first to second five-minute interval (*p* = 0.025).

### 3.3. Valence and Arousal Scale

Inter-rater reliability analysis demonstrated that agreement was moderate for the Valence scale (ICC: 0.64) but was poor for the Arousal scale (ICC: 0.28). Intra-rater reliability was good for all raters for valence (ICC: 0.78–0.89) but was poor for arousal for Coder 1 (ICC: 0.36) and Coder 2 (ICC: 0.23) and moderate for Coder 3 (ICC: 0.74) and Coder 4 (ICC: 0.61).

Based on these reliability results, Coder 1 and 2 were removed from the analysis of the Valence and Arousal scales. In addition, an average score of the two remaining coders was used for analysis to minimize the impact of inter-rater variation. With these modifications, the Valence and Arousal scales were considered sufficiently reliable for analysis.

**Figure 8:**
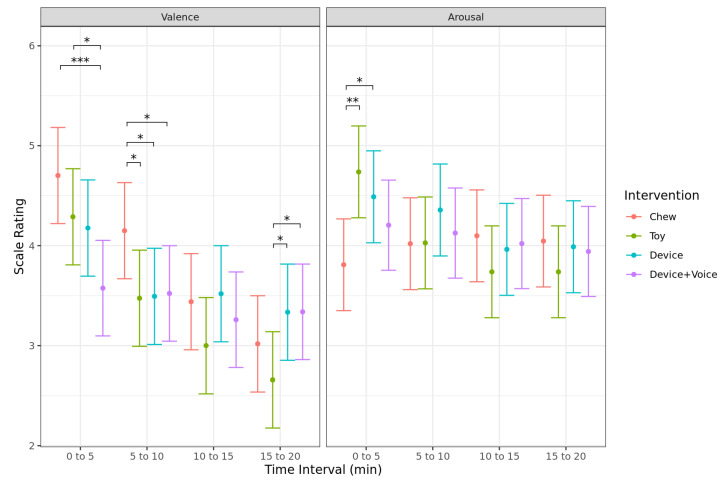
Predicted mean (95% CI) scores for the Valence scale and Arousal scale for each 5 min interval when dogs were left alone with one of four enrichments for 20 min. Asterisks indicate significant differences between treatment groups within each time interval. Three asterisks indicate significance at *p*  <  0.001, two asterisks indicate significance at *p*  <  0.01, and one asterisk indicates significance at *p*  <  0.05.

There was a significant interaction between time and intervention for the Valence scale (*p* < 0.001; Figure 8). Pairwise comparisons identified that the Chew had significantly higher Valence scores (indicating positive valence) compared to the Device + Voice (*p* < 0.001) intervention during the first five minutes, and compared to the Toy (*p* = 0.020), Device (*p* = 0.026), and Device + Voice (*p* = 0.038) interventions during the second five minutes. Additionally, the Toy had significantly higher Valence scores compared to the Device + Voice (*p* = 0.013) intervention during the first five minutes, and lower Valence scores compared to the Device (*p* = 0.020) and Device + Voice (*p* = 0.020) interventions during the last five minutes. There were no other significant differences between the interventions during the last 10 min of the test session. The Chew decreased Valence scores significantly from the second five minutes to the third five minutes (*p* = 0.013) but did not change between any other consecutive time interval. The Toy decreased Valence scores significantly from the first five minutes to the second five minutes (*p* = 0.003) but did not change between the remaining time intervals. Similarly, the Device intervention decreased Valence scores significantly from the first five minutes to the second five minutes (*p* = 0.018) but did not change between the remaining time intervals. The Device + Voice intervention did not significantly change Valence scores between any time interval.

There was a significant interaction between time and intervention for the Arousal scale (*p* = 0.020; Figure 8). Pairwise comparisons identified that the Chew had significantly lower Arousal scores (indicating calm responses) compared to the Toy (*p* = 0.001) and Device (*p* = 0.028) interventions during the first five minutes. There were no other significant differences in Arousal scores between the interventions at any other timepoint. The Toy showed a decrease in Arousal scores from the first five minutes to the second five minutes (*p* = 0.019) but did not change between the remaining time intervals. No other intervention showed a significant change in Arousal scores across time.

## 4. Discussion

The aims of the present study were to identify which forms of enrichment dogs chose to interact with when left alone and what effect these enrichments had on their emotional state. Engagement and emotional states were measured in this study using a combination of objectively defined and coded behaviors, and more subjective ratings using a QBA and scales for Valence and Arousal levels.

Objectively defined and coded behaviors are a valuable tool that can be used for evaluating emotional states and welfare in animals [32]. Destructive and escape behaviors are commonly reported by owners of dogs with separation-related problems, and may be reflective of frustration that is either directed at the exit, or re-directed on other objects in the room [2]. Similarly, inappropriate elimination, pacing, and restlessness are commonly reported separation-related behaviors and may be indicative of dogs experiencing negative emotional states during social isolation [2]. In the current study, these behaviors were not frequently observed, indicating a majority of the dogs tested likely experienced mild-to-moderate levels of stress. This is supported by the ratings from the QBA and Valence scale typically being close to neutral. While destructive behaviors were observed in some of the dogs, these were primarily digging/scratching at the bed or crate that were likely attempts to obtain inaccessible treats/toys.

While more subjective than coded behaviors, the QBA is a useful tool that provides a holistic view of how an animal may be feeling, is generally quick to administer, and is more efficient than coding a series of specific behaviors [33]. This tool has previously been established as being accurate and reliable for use in measuring behavior in a range of species [33,34,35,36], including dogs [26,30,37,38,39]. In the current study, analysis of a refined list of terms identified two primary components of interest: ‘Stressed/Anxious’ and ‘Interactive’. The ‘Stressed/Anxious’ component is similar to components identified in previous research (e.g., ‘PC2′ [26,39] and ‘Stressed/Anxious’. [38]), with high loadings for terms indicative of negative emotional states, such as ‘anxious’ and ‘stressed’ in one direction, and for terms indicative of positive emotional states, such as ‘comfortable’ and ‘relaxed’ in the other. Similarly, the ‘Interactive’ component from the present study was comparable to components identified in previous research (e.g., ‘PC1′ [26,39] and ‘Interactive/Engaged’ [38]), with high loadings for terms indicative of interactions, such as ‘curious’ and ‘explorative’. The term ‘frustrated’ also loaded on this component in the present study. While this term was not used in previous research identifying ‘Interactive’ components, it is not unexpected it would load strongly. Frustration was likely elicited in dogs that were more interactive when they no longer had direct access to the interventions, or when their interactions failed to have the anticipated results. While some concerns with reliability were initially observed with particular terms, both inter- and intra-rater reliability were deemed acceptable using the refined component scores and were similar to those reported previously in other studies [26,30,38,39]. This highlights the need for the robust training and assessment of reliability of coders, while demonstrating that QBAs provide useful holistic insights into how animals might be feeling.

Ratings of valence and arousal were also used to provide a holistic impression of the dogs’ emotional state. These scales have been successfully used to evaluate the emotional state of dogs from photos [40], but to the authors’ knowledge, they have not been used to assess the emotional states of dogs from videos. Additionally, factors such as personality and previous dog experience can influence a person’s ratings of valence and arousal [40]. The reliability results from the current study indicated there was poor agreement for the Arousal scale, highlighting the need for additional training and, potentially, more detailed definitions to avoid differences in the interpretation of this term between coders. Alternatively, different methods of measuring valence and arousal could be used, such as the utilization of a pictorial scale [41] or allowing raters to select a point on a coordinate system [42]. However, acceptable levels of reliability were able to be achieved during the current study following the removal of two coders.

Considering the results using data from reliable coders and from all measures, dogs with the long-lasting chew were rated as being the least stressed/anxious, and having the most positive low-arousal emotional responses, and dogs with the food-dispensing toy were rated as being interactive and high arousal, while the dogs were actively engaging with the enrichments. However, once engagement ceased, either through the consumption of all the food or the enrichment becoming inaccessible, emotional responses were rated as more neutral or negative. The responses to the smart food-dispensing device were rated as more neutral and were relatively consistent over time. It should be noted that there was no control intervention used in the current study, so it is unknown how these emotional responses would compare to social isolation events with no enrichment present. Additionally, the volume and palatability of food offered was not the same across the different interventions, which may have impacted the dogs depending on their personal preference and/or level of hunger. In this study, the chew and toy were given to the dogs at the beginning of the event, which may have distracted them from realizing they were alone in the room. It is possible these longer-lasting enrichments may not relieve stress, but instead distract dogs from the stressor with a positive stimulus. Humans use self-distraction as a coping strategy to deal with negative life events, such as anxiety [43,44,45], and dogs may do something similar. A recent study found that food-based enrichments were successful in alleviating problematic behaviors in dogs with separation anxiety, both while the dog was interacting with the enrichments and for 15 min after the enrichments were removed [25]. However, it is still unknown what effect these short-term positive emotions would have on the dog’s emotional state during longer-term periods of social isolation.

During the current trial, the long-lasting chew was rated as having less stressed/anxious emotions and more positive and low-arousal emotions, especially during the first five to ten minutes of the session. This aligns with the average duration of engagement with the chew that was observed being approximately nine minutes. The positive low-arousal emotional ratings identified during this trial also support recent survey data that showed dog owners believed chewing was calming for their dogs and prevented boredom [46]. The same study found negative high-arousal events were moderately correlated with chewing behavior in dogs across all age groups [46], suggesting these behaviors may aid individuals when dealing with negative emotions. Similar stress-relieving effects of chewing have been shown to occur in humans [47,48]; however, there is little empirical evidence of this effect in dogs, despite recommendations by pet professionals to utilize chewing behavior for stress relief [49]. Interestingly, the dogs were scored as being significantly less interactive, as measured with the QBA, during the first five minutes with the chew, than with the other interventions. This is likely due to the specific terms that loaded strongly on this component (e.g., curious, explorative, and frustrated) being more reflective of active engagement and exploration rather than calm engagement and consumption. It should be noted that the manufacturer of the chew used during the current trial recommends against unsupervised use. While no negative interactions were observed during the current study, potential safety concerns should be considered in future research and when making recommendations for use with dogs in a home environment. Further research investigating chew-based enrichments would be beneficial to understanding their safety and impact on emotional wellbeing during longer periods of social isolation.

During the current trial, the food-dispensing toy resulted in higher arousal and interactive ratings compared to the chew during the first five minutes of the event, which was also the average duration of engagement with this enrichment. This is likely due to the positive anticipation related to prior learning of treats being dispensed from the toy, as well as the active ‘play’-type behaviors that are required for the toy to dispense treats. However, during the last 10 min of the event, the toy was scored as less interactive compared to the devices and also resulted in more negative valence ratings, frequently following the toy becoming empty or stuck. There were eleven instances (58%) where the toy became lodged under the elevated dog bed present in the room for at least a portion of the test session. This inaccessibility of expected treats, including instances when the toy ran out of treats, may have induced negative emotional states such as frustration or sadness. Surprising reward omission is known to lead to negative emotional states, especially frustration, in many species including dogs [50,51]. Additionally, signs of frustration have been observed in dogs in another study when enrichments malfunctioned [52], turning positive anticipation into frustration. Therefore, measures should be taken to ensure enrichments are always accessible to dogs (i.e., block areas where enrichments may get stuck) or are only used when supervised. Additionally, clear indicators that the enrichment is empty would likely be beneficial, so the dog does not have expectations of continued treat delivery. It is also possible that dogs with higher arousal levels were more likely to have vigorous interactions with the toy resulting in the toy becoming stuck. It should be noted that out of the eight instances where the toy did not become stuck, for four of them (50%), the dog failed to meaningfully engage with the enrichment. This was the only enrichment that some dogs failed to meaningfully engage with, and these dogs all successfully interacted with the toy in the presence of a handler. This may indicate that the dogs are less motivated to work to obtain food from the toy when experiencing the stress of social isolation. This effect may have been amplified by the use of main meal kibble for this intervention, which may have been less palatable to the dogs than the chew. Numerous studies using rats as a model for mammalian brain structure and function have indicated that acute stress and the anticipation of negative events result in a reduction in motivation to eat and food intake (i.e., anorexia) [53]. A study by Kang [25] found that food-based enrichments which required dogs to work to obtain the food were successful in alleviating problem behaviors related to separation from their owner. However, it should be noted that a researcher was present in the room throughout testing in that study, so while dogs were separated from their caregiver, it did not include complete social isolation. These results highlight the need for enrichments to be carefully selected to encourage engagement even during periods of mild stress, but also ensure that negative emotional states are not inadvertently replaced with other, potentially worse, negative emotions. Additionally, as with the chew, the manufacturer of the toy recommends against unsupervised use. Therefore, potential safety concerns should be considered in future research and when making recommendations for use with dogs in a home environment. Based on these results, this specific toy enrichment may be better utilized as a form of supervised mental stimulation rather than a tool to aid in alleviating negative emotional states in dogs during separation from their owner.

During the current study, the smart food-dispensing device had higher ratings for stressed/anxious responses and lower ratings for positive emotional responses in comparison to the chew during the first five to ten minutes of the event. However, the devices had higher ratings for positive emotions and interaction during the last ten minutes compared to the toy. This may have been due to the intermittent nature of treat dispersal not being sufficient to distract the dog during the beginning of the event, but meant treats continued to be dispensed consistently throughout the event, unlike other interventions where food was commonly consumed during the first half of the session. For the purposes of consistency and to allow time for the voice script to be read, the treats were delivered at one-minute intervals throughout the event. However, this form of treat delivery is most likely not reflective of how an owner would use the device to interact with their dog during extended periods of separation. It is possible that more frequent delivery, especially targeted around periods where the dog is showing signs of stress, may be more beneficial. Conversely, having longer periods of time without treat delivery may also allow dogs to settle and rest without disturbance. In addition, while all the dogs were exposed to the device before testing, this was still a novel form of treat delivery for the dogs, and they had never been exposed to the device without a handler present. This may have resulted in a novelty effect, with some dogs being startled by the food appearance or the sound of the voice. Further exploration of this device used in a more realistic home setting, with more prior conditioning and in a manner more reflective of how it would be used by dog owners, would be beneficial.

During the study, the addition of a voice recording to the device throwing treats did not make a difference to engagement or ratings of the dog’s emotional state. In the current study, voice recordings were made by experimenters who had limited familiarity with the individual dogs. It has previously been shown that dogs can recognize a recording of their owner’s voice in comparison to a stranger [54], and it is, therefore, possible that the lack of familiarity in the voice used in the current study may have impacted the results. Another study by Tiira [55] found owner voice recordings reduced vocalizations in dogs when left at home alone, implying voice recordings are relevant to pets. However, it is unclear if the voice recordings used by Tiira [55] had a positive or negative effect on the emotional wellbeing of the pets. Previous research on dogs in shelter environments indicate that exposure to audiobooks results in more time spent resting and less time vocalizing in comparison to a control condition or different types of music [56]. The authors hypothesize that the focused nature of speech delivery in audiobooks, as compared to general conversation, may contribute to the dogs responding positively to the recording [56]. Consequently, it is possible that voice familiarity in the current study may not have been the sole reason for the lack of observed effects, and the manner in which the speech was delivered may also be important. Further research into the relevance that different aspects of voice recordings have to dogs would be beneficial in understanding how a human voice can be used to improve dog emotional wellbeing.

This study was a first exploration into how dogs interact with different food-based enrichments when experiencing the stressor of short-term social isolation, and some limitations should be considered. Primarily, the population of dogs consisted of only two breeds, and comprised dogs housed within a research institute rather than privately owned pet dogs. While the test room was a familiar area to the dogs, it was not their home environment, which may have contributed additional stress. These dogs also have very limited exposure to complete social isolation as they are pair- or group-housed, and typically only leave their kennel when in the presence of a handler. They may, therefore, find periods of social isolation more stressful than a pet dog. The dogs also had very limited exposure to the enrichments used, which may have resulted in novelty effects and impacted their emotional responses. In addition, the period of isolation used for this study was very short in comparison to what is normally experienced by dogs in a home environment. Further investigation into the use of these enrichments in a more realistic setting is warranted.

## 5. Conclusions

Overall, this study was successful in identifying food-based enrichments that dogs engage with during short periods of social isolation. The long-lasting chew was found to improve emotional states in comparison to the other interventions tested, based on greater time spent engaged, lower ratings for stressed/anxious responses, and higher ratings for positive and low-arousal emotional responses. Further investigation into this type of enrichment is recommended, including assessment of the effect on emotional wellbeing in comparison to a no-enrichment control, during a longer-duration social isolation event and/or with a population of pet dogs in a home environment. Additionally, comparisons of different types of long-lasting chews and their impact on dog behavior and emotional wellbeing would be beneficial.

## Figures and Tables

**Table 1:** Ethogram used for the coding of behavioral measures from videos.

Behavior	Definition	Type
Engaging with enrichment (contact)	Exploring and interacting with enrichment and/or treats dispensed from the enrichment with physical contact. Includes touching with muzzle/paw and consuming.	State
Engaging with enrichment (no contact)	Exploring and interacting with enrichment and/or treats dispensed from the enrichment without physical contact. Includes looking at, sniffing, and circling enrichment and/or treats dispensed from the enrichment.	State
Other activity	Movement around the room, which can include repositioning, excluding any other codable behavior.	State
Other stationary	Not moving around the room, excluding any other codable behavior.	State
Escape	Tries to dig/bite/scratch the external door/retractable gate—not directed at themselves or anything else in the room.	State
Destructive	Mouth/front paws and claws used to attempt movement/displacement of substrate other than external door/retractable gate (e.g., dog bed/bedding). Not directed at themselves or anything else in the room.	State
Autogrooming	Bites/scratches/licks their own body.	State
Elimination	Squat/leg raise to urinate and/or hind end lowered and back arched in order to defecate.	Event
Shake-off	Head and/or full body is consciously shaken.	Event

**Table 2:** Qualitative Behavioral Assessment (QBA) used to measure dog behavior at 5 min intervals during test sessions.

Term	Definition
Anxious	Worried, unable to settle or cope with the environment, apprehensive
Alert	Vigilant, inquisitive, on guard [26]
Bored	Disinterested, passive, showing suboptimal arousal levels/drowsiness signs [26]
Comfortable	Without worries, settled in the environment, peaceful with external stimuli [26] *
Curious	Actively interested in people or things, explorative, inquiring in a positive relaxed manner [26]
Depressed	Dull, sad demeanor; disengaged from and unresponsive to the environment, quiet, apathetic [26]
Excited	Positively agitated in response to external stimuli, euphoric, exuberant, thrilled [26]
Explorative	Confident in exploring the environment or new stimuli, investigative
Fearful	Timid, scared, shows postures typical of fear [26] *
Hesitant	Unsure, doubtful, shows conflicting behavior, uncertain whether to approach or trust a stimulus [26] *
Interested	Attentive, attracted to stimuli and attempts to approach them [26]
Nervous	Uneasy, agitated, shows fast arousal, unsettled, restless, hyperactive [26]
Playful	Cheerful, high spirits, fun, shows play-related behavior [26] *
Reactive	Responsive to external stimuli [26]
Relaxed	Easy-going, calm with no visual evidence of tension in the body [26] *
Stressed	Tense, shows signs of distress [26]
Calm	Absence of strong positive/negative emotions
Engaged	Actively focused on a specific object or task, attempts to interact with object
Frustrated	Annoyed, irritable, restless, unable to obtain what it wants, impatient
Lethargic	Sluggish, inactive, unresponsive, or slow to respond to external stimuli
Restless	Unable to rest or relax
Sad	Unhappy, downcast
Tense	Stiff, rigid posture, on edge
Uncomfortable	Uneasy, nervous, tense, restless

* definition modified from original source.

**Table 3:** Intraclass correlation coefficients indicating levels of agreement both between coders (inter-rater reliability) and within each of the four coders (intra-rater reliability) for each term of the QBA.

Term	Inter	Intra			
		Coder 1	Coder 2	Coder 3	Coder 4
Anxious	0.65	0.83	0.99	0.77	0.61
Alert	0.41	0.24	0.97	0.60	0.78
Bored	0.67	0.05	0.94	0.75	-
Calm	0.56	0.79	0.97	0.56	0.42
Comfortable	0.65	0.69	0.98	0.87	0.61
Curious	0.53	0.70	0.99	0.58	0.42
Depressed	0.65	0.45	0.97	0.84	-
Engaged	0.45	0.58	1.00	0.94	0.65
Excited	0.66	0.42	0.98	0.90	0.11
Explorative	0.34	0.67	0.97	0.77	0.52
Fearful	0.79	0.72	0.65	−0.01	-
Frustrated	0.47	0.66	0.84	0.91	0.49
Hesitant	0.72	0.68	0.79	0.74	0.77
Interested	0.48	0.49	1.00	0.87	0.80
Lethargic	0.59	0.40	-	−0.09	-
Nervous	0.64	0.49	0.99	0.36	0.26
Playful	0.45	0.13	0.92	0.40	0.00
Reactive	0.62	0.38	0.97	0.88	0.75
Relaxed	0.60	0.72	0.95	0.76	0.65
Restless	0.50	0.90	0.96	0.75	0.66
Sad	0.70	0.52	0.83	0.61	−0.02
Stressed	0.66	0.64	0.95	0.75	0.36
Tense	0.70	0.59	0.99	0.23	0.54
Uncomfortable	0.70	0.68	0.95	0.72	0.59

**Table 4:** Components extracted by the PCA of QBA scores. Loadings≥ |0.50| are in bold.

Term	PC1-Stressed/Anxious	PC2-Interactive
Uncomfortable	**0.93**	−0.02
Anxious	**0.92**	0.03
Stressed	**0.91**	0.12
Sad	**0.63**	−0.09
Restless	**0.61**	0.46
Hesitant	**0.52**	−0.13
Comfortable	**−0.90**	0.09
Relaxed	**−0.86**	−0.19
Calm	**−0.81**	−0.28
Engaged	**−0.57**	0.48
Curious	−0.48	**0.66**
Frustrated	0.47	**0.50**
Explorative	−0.33	**0.71**

## Data Availability

The data that support the findings of this study are available from the corresponding authors upon reasonable request.

## Appendix A. Device Script

Hello [Dog Name], are you being a good [boy/girl]? Would you like a treat?

I’m just at work right now, but I’ll be back soon. How are you doing [Dog Name]?

As soon as I’m back we can go for a walk together, let’s go to Rose’s Garden in a bit!

Guess what, I’ve got some more treats for you! Coming your way now!

I heard you got a [Last Enrichment] the last time you came here [Dog Name]. I wonder if you like the [Device] better?

What are you up to [Dog Name]? Hope you’re having fun doing dog stuff.

You’re such a beautiful [girl/boy], I wish I was with you right now to have a cuddle.

I’ll be coming back for you soon, not too long to wait. You’re being such a good dog!

I just saw your friend [Other Dog Name] not too long ago. She said that she can’t wait to have a run around in the play area with you later.

Hmm, I wonder what [Dog Name] is doing, looks like you are okay! Have some biscuits from me.

We’re halfway through [Dog Name], not long to go now! You’re being a good [boy/girl]!

Ahhh you’re so [cute/handsome/fluffy/beautiful]! When I get back we can have a snuggle on the sofa.

I wonder what else we could do later when I’m home…we can play with a toy or go for a run? Whatever you’d like to do!

You’re being such a good [girl/boy] [Dog Name], here’s some treats for you.

I hope you’re enjoying your new biscuits! Are they tasty?

Are you enjoying the [Device] [Dog Name]? Here’s a treat!

The weather today looks sunny, I hope you’re keeping warm! You could always snuggle up in the vet bed if you get cold.

[Dog Name], here comes some more biscuits!

Let’s play a game, when I finish talking… treats will pop out! Are you ready? One… two… three!

Are you ready for your Mum to pick you up now? Just one more minute! I hope you’ve had a good time.

## References

[1] Prato-Previde E., Custance M.E., Spiezio C., Sabatini F. (2003). Is the Dog-Human Relationship an Attachment Bond? An Observational Study Using Ainsworth’s Strange Situation. Behaviour.

[2] De Assis L.S., Matos R., Pike T.W., Burman O.H.P., Mills D.S. (2020). Developing Diagnostic Frameworks in Veterinary Behavioral Medicine: Disambiguating Separation Related Problems in Dogs. Front. Vet. Sci..

[3] Rehn T., Keeling L.J. (2011). The Effect of Time Left Alone at Home on Dog Welfare. Appl. Anim. Behav. Sci..

[4] Applebaum J.W., Tomlinson C.A., Matijczak A., McDonald S.E., Zsembik B.A. (2020). The Concerns, Difficulties, and Stressors of Caring for Pets during COVID-19: Results from a Large Survey of U.S. Pet Owners. Animals.

[5] Harvey N.D., Christley R.M., Giragosian K., Mead R., Murray J.K., Samet L., Upjohn M.M., Casey R.A. (2022). Impact of Changes in Time Left Alone on Separation-Related Behaviour in UK Pet Dogs. Animals.

[6] Butler R., Sargisson R.J., Elliffe D. (2011). The Efficacy of Systematic Desensitization for Treating the Separation-Related Problem Behaviour of Domestic Dogs. Appl. Anim. Behav. Sci..

[7] Gaultier E., Bonnafous L., Bougrat L., Lafont C., Pageat P. (2005). Comparison of the Efficacy of a Synthetic Dog-Appeasing Pheromone with Clomipramine for the Treatment of Separation-Related Disorders in Dogs. Vet. Rec..

[8] King J.N., Simpson B.S., Overall K.L., Appleby D., Pageat P., Ross C., Chaurand J.P., Heath S., Beata C., Weiss A.B. (2000). Treatment of Separation Anxiety in Dogs with Clomipramine: Results from a Prospective, Randomized, Double-Blind, Placebo-Controlled, Parallel-Group, Multicenter, Clinical Trial. Appl. Anim. Behav. Sci..

[9] Karagiannis C.I., Burman O.H.P., Mills D.S. (2015). Dogs with Separation-Related Problems Show a “Less Pessimistic” Cognitive Bias during Treatment with Fluoxetine (Reconcile^TM^) and a Behaviour Modification Plan. BMC Vet. Res..

[10] Landsberg G.M., Melese P., Sherman B.L., Neilson J.C., Zimmerman A., Clarke T.P. (2008). Effectiveness of Fluoxetine Chewable Tablets in the Treatment of Canine Separation Anxiety. J. Vet. Behav..

[11] Ogata N. (2016). Separation Anxiety in Dogs: What Progress Has Been Made in Our Understanding of the Most Common Behavioural Problems in Dogs?. J. Vet. Behav..

[12] Sargisson R. (2014). Canine Separation Anxiety: Strategies for Treatment and Management. Vet. Med. Res. Reports.

[13] Newberry R.C. (1995). Environmental Enrichment: Increasing the Biological Relevance of Captive Environments. Appl. Anim. Behav. Sci..

[14] Tarou L.R., Bashaw M.J. (2007). Maximizing the Effectiveness of Environmental Enrichment: Suggestions from the Experimental Analysis of Behavior. Appl. Anim. Behav. Sci..

[15] Mason G.J., Clubb R., Latham N., Vickery S. (2007). Why and How Should We Use Environmental Enrichment to Tackle Stereotypic Behaviour?. Appl. Anim. Behav. Sci..

[16] Swaisgood R.R., Shepherdson D.J. (2005). Scientific Approaches to Enrichment and Stereotypies in Zoo Animals: What’s Been Done and Where Should We Go Next?. Zoo Biol..

[17] Godyn D., Nowicki J., Herbut P. (2019). Effects of Environmental Enrichment on Pig Welfare—A Review. Animals.

[18] Riber A.B., van de Weerd H.A., de Jong I.C., Steenfeldt S. (2018). Review of Environmental Enrichment for Broiler Chickens. Poult. Sci..

[19] Wells D.L. (2004). A Review of Environmental Enrichment for Kennelled Dogs, Canis Familiaris. Appl. Anim. Behav. Sci..

[20] Hubrecht R.C. (1993). A Comparison of Social and Environmental Enrichment Methods for Laboratory Housed Dogs. Appl. Anim. Behav. Sci..

[21] Schipper L., Vinke C.M., Schilder M.B.H., Sprujit B.M. (2008). The Effect of Feeding Enrichment Toys on the Behaviour of Kenneled Dogs (Canis Familiaris). Appl. Anim. Behav. Sci..

[22] Herron M.E., Kirby-Madden T.M., Lord L.K. (2014). Effects of Environmental Enrichment on the Behavior of Shelter Dogs. J. Am. Vet. Med. Assoc..

[23] Kogan L.R., Schoenfeld-Tacher R., Simon A.A. (2012). Behavioral Effects of Auditory Stimulation on Kenneled Dogs. J. Vet. Behav..

[24] Hunt R.L., Whiteside H., Prankel S. (2022). Effects of Environmental Enrichment on Dog Behaviour: Pilot Study. Animals.

[25] Kang O.-D. (2022). Effects of Environment Enrichment on Behavioral Problems in Dogs with Separation Anxiety. J. Environ. Sci. Int..

[26] Arena L., Wemelsfelder F., Messori S., Ferri N., Barnard S. (2019). Development of a Fixed List of Terms for the Qualitative Behavioural Assessment of Shelter Dogs. PLoS ONE.

[27] Kujala M.V., Somppi S., Jokela M., Vainio O., Parkkonen L. (2017). Human Empathy, Personality and Experience Affect the Emotion Ratings of Dog and Human Facial Expressions. PLoS ONE.

[28] Koo T.K., Li M.Y. (2016). A Guideline of Selecting and Reporting Intraclass Correlation Coefficients for Reliability Research. J. Chiropr. Med..

[29] Ferguson E., Cox T. (1993). Exploratory Factor Analysis: A Users’ Guide. Int. J. Sel. Assess..

[30] Shaw N., Wemelsfelder F., Riley L.M. (2022). Bark to the Future: The Welfare of Domestic Dogs during Interaction with a Positively Reinforcing Artificial Agent. Appl. Anim. Behav. Sci..

[31] R Core Team (2022). R: A Language and Environment for Statistical Computing. R Foundation for Statistical Computing, Vienna, Austria. https://www.R-project.org/,.

[32] Dawkins M.S. (2003). Behaviour as a Tool in the Assessment of Animal Welfare. Zoology.

[33] Browning H. (2022). Assessing Measures of Animal Welfare. Biol. Philos..

[34] Fleming P.A., Clarke T., Wickham S.L., Stockman C.A., Barnes A.L., Collins T., Miller D.W. (2016). The Contribution of Qualitative Behavioural Assessment to Appraisal of Livestock Welfare. Anim. Prod. Sci..

[35] Travnik I.C., Machado D.S., Sant’Anna A.C. (2022). Do You See the Same Cat That I See? Inter-and Intra-Observer Reliablity for Qualitative Behaviour Assessment as Temperament Indicator in Domestic Cats. Anim. Welf..

[36] Cooke A.S., Mullan S.M., Morten C., Hockenhull J., Lee M.R.F., Cardenas L.M., Rivero M.J. (2022). V-QBA vs. QBA—How Do Video and Live Analysis Compare for Qualitative Behaviour Assessment?. Front. Vet. Sci..

[37] Walker J.K., Dale A.R., D’Eath R.B., Wemelsfelder F. (2016). Qualitative Behaviour Assessment of Dogs in the Shelter and Home Environment and Relationship with Quantitative Behaviour Assessment and Physiological Responses. Appl. Anim. Behav. Sci..

[38] King T., Flint H.E., Hunt A.B.G., Werzowa W.T., Logan D.W. (2022). Effect of Music on Stress Parameters in Dogs during a Mock Veterinary Visit. Animals.

[39] Stubsjøen S.M., Moe R.O., Bruland K., Lien T., Muri K. (2020). Reliability of Observer Ratings: Qualitative Behaviour Assessments of Shelter Dogs Using a Fixed List of Descriptors. Vet. Anim. Sci..

[40] Kujala M.V., Kujala J., Carlson S., Hari R. (2012). Dog Experts’ Brains Distinguish Socially Relevant Body Postures Similarly in Dogs and Humans. PLoS ONE.

[41] Singh A., Young J.E., Kotze P., Marsden G., Lingaard G., Wesson J., Winckler M. (2013). A Dog Tail for Utility Robots: Exploring Affective Properties of Tail Movement. Proceedings of the Human-Computer Interaction–INTERACT 2013: 14th IFIP TC 13 International Conference, Cape Town, South Africa, 2–6 September 2013.

[42] Faragó T., Andics A., Devecseri V., Kis A., Gácsi M., Miklósi Á. (2014). Humans Rely on the Same Rules to Assess Emotional Valence and Intensity in Conspecific and Dog Vocalizations. Biol. Lett..

[43] Kraaij V., Garnefski N. (2019). The Behavioral Emotion Regulation Questionnaire: Development, Psychometric Properties and Relationships with Emotional Problems and the Cognitive Emotion Regulation Questionnaire. Pers. Individ. Dif..

[44] Schreiber K.L., Campbell C., Martel M.O., Greenbaum S., Wasan A.D., Borsook D., Jamison R.N., Edwards R.R. (2014). Distraction Analgesia in Chronic Pain Patients: The Impact of Catastrophizing. Anesthesiology.

[45] Tuncay T., Musabek I., Gok D.E., Kutlu M. (2008). The Relationship between Anxiety, Coping Strategies and Characteristics of Patients with Diabetes. Health Qual. Life Outcomes.

[46] Arhant C., Winkelmann R., Troxler J. (2021). Chewing Behaviour in Dogs—A Survey-Based Exploratory Study. Appl. Anim. Behav. Sci..

[47] Scholey A., Haskell C., Robertson B., Kennedy D., Milne A., Wetherell M. (2009). Chewing Gum Alleviates Negative Mood and Reduces Cortisol during Acute Laboratory Psychological Stress. Physiol. Behav..

[48] Tasaka A., Tahara Y., Sugiyama T., Sakurai K. (2008). Influence of Chewing Rate on Salivary Stress Hormone Levels. Nihon Hotetsu Shika Gakkai Zasshi.

[49] Rooney N., Gaines S., Hiby E. (2009). A Practitioner’s Guide to Working Dog Welfare. J. Vet. Behav..

[50] Jakovcevic A., Elgier A.M., Mustaca A.E., Bentosela M. (2013). Frustration Behaviors in Domestic Dogs. J. Appl. Anim. Welf. Sci..

[51] Papini M.R., Dudley R.T. (1997). Consequences of Surprising Reward Omissions. Rev. Gen. Psychol..

[52] Wallis L.J., Range F., Kubinyi E., Chapagain D., Serra J., Huber L. Utilising Dog-Computer Interactions to Provide Mental Stimulation in Dogs Especially during Ageing. Proceedings of the Fourth International Conference on Animal-Computer Interaction.

[53] Petrovich G.D. (2013). Forebrain Networks and the Control of Feeding by Environmental Learned Cues. Physiol. Behav..

[54] Adachi I., Kuwahata H., Fujita K. (2006). Dogs Recall Their Owner’s Face upon Hearing the Owner’s Voice. Anim. Cogn..

[55] Tiira K. (2021). Digital Dogsitter^®^ Reduces Vocalization in Dogs Suffering from Separation-Related Problems. Appl. Anim. Behav. Sci..

[56] Brayley C., Montrose V.T. (2016). The Effects of Audiobooks on the Behaviour of Dogs at a Rehoming Kennels. Appl. Anim. Behav. Sci..

